# Prohibitin overexpression improves myocardial function in diabetic cardiomyopathy

**DOI:** 10.18632/oncotarget.6384

**Published:** 2015-11-25

**Authors:** Wen-qian Dong, Min Chao, Qing-hua Lu, Wei-li Chai, Wei Zhang, Xue-ying Chen, Er-shun Liang, Ling-bo Wang, Hong-liang Tian, Yu-guo Chen, Ming-xiang Zhang

**Affiliations:** ^1^ The Key Laboratory of Cardiovascular Remodeling and Function Research, Chinese Ministry of Education and Chinese Ministry of Public Health, Qilu Hospital, Shandong University, Jinan, China; ^2^ Department of Cardiology, The Second Hospital of Shandong University, Jinan, China; ^3^ Department of Emergency, Qilu Hospital, Shandong University, Jinan, China; ^4^ Department of Cardiology, The Third Hospital of Jinan, Shandong, China; ^5^ Department of Anorectal Surgery, Affiliated Hospital of Jining Medical College, Jining, Shandong, China

**Keywords:** prohibitin, myocardial fibrosis, apoptosis, diabetic cardiomyopathy, Pathology Section

## Abstract

Prohibitin (PHB) is a highly conserved protein implicated in various cellular functions including proliferation, apoptosis, tumor suppression, transcription, and mitochondrial protein folding. However, its function in diabetic cardiomyopathy (DCM) is still unclear. *In vivo*, type 2 diabetic rat model was induced by using a high-fat diet and low-dose streptozotocin. Overexpression of the PHB protein in the model rats was achieved by injecting lentivirus carrying PHB cDNA via the jugular vein. Characteristics of type 2 DCM were evaluated by metabolic tests, echocardiography and histopathology. Rats with DCM showed severe insulin resistance, left ventricular dysfunction, fibrosis and apoptosis. PHB overexpression ameliorated the disease. Cardiofibroblasts (CFs) and H9c2 cardiomyoblasts were used *in vitro* to investigate the mechanism of PHB in altered function. In CFs treated with HG, PHB overexpression decreased expression of collagen, matrix metalloproteinase activity, and proliferation. In H9c2 cardiomyoblasts, PHB overexpression inhibited apoptosis induced by HG. Furthermore, the increased phosphorylation of extracellular signal–regulated kinase (ERK) 1/2 was significantly decreased and the inhibited phosphorylation of Akt was restored in DCM. Therefore, PHB may be a new therapeutic target for human DCM.

## INTRODUCTION

Diabetic cardiomyopathy (DCM) is diagnosed when ventricular dysfunction develops in patients with diabetes who do not exhibit coronary atherosclerosis and hypertension [1]. It is one of the major cardiac complications in diabetic patients [2]. Left ventricular (LV) systolic and diastolic dysfunction, LV hypertrophy, and alterations in the coronary microcirculation are all observed in DCM [3]. Notable modifications in the structure of the diabetic heart include excess deposition of extracellular matrix (ECM) proteins and increased cardiomyocyte apoptosis, which lead to fibrosis and have been implicated in the development of dilated DCM [4–6]. Chronic hyperglycemia is one of the pathophysiological stimuli involved in the tissue injury and diastolic dysfunction of DCM [7].

Prohibitin (PHB) is a highly conserved and pleiotropic protein that is ubiquitously expressed in various compartments of eukaryocytes including the mitochondria, nucleus, and plasma membrane [8]. It is implicated in various cellular functions including proliferation, apoptosis, tumor suppression, transcription, and mitochondrial protein folding [9]. The most well characterized feature is its role as a mitochondrial chaperone [10]. Lack of PHB is associated with mitochondrial membrane depolarization and increased generation of reactive oxygen species (ROS) that are often related to apoptosis [11, 12]. PHB expression has been proven to be negatively correlated with the liver and renal interstitial fibrosis [13, 14]. In addition, Supale et al. have reported that mouse with PHB deficiency in β cells showed lower release of insulin because of reduced β cell mass, which led to abnormal glucose intolerance [15]. While many studies have examined the effects of PHB, little is known about the function of this protein in DCM and nothing has been reported about the function of PHB in diabetic rat heart.

Previous studies have demonstrated that mitogen-activated protein kinases (MAPKs) and the Akt signal pathway are closely associated with PHB in cell-cycle regulation, apoptosis, and inflammation [16–18]. However, the role of the PHB/MAPK and PHB/Akt signal transduction pathways in the progression of DCM has not been investigated.

We hypothesized that PHB overexpression may have a protective effect on the myocardium in diabetes. To uncover the mechanisms of PHB in DCM, we investigated its role *in vitro* in neonatal cardiofibroblasts (CFs) and H9c2 cardiomyoblasts induced with HG and *in vivo* in a DCM rat model.

## RESULTS

### Characteristics of diabetic rats

**Figure 1:**
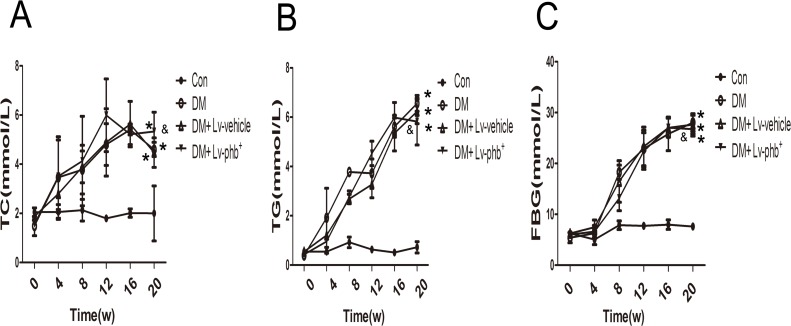
Basic characteristics of rats **A**. Total cholesterol (TC) level. **B**. Triglyceride (TG) level. **C**. Fasting blood glucose (FBG) level. Con: normal rats, DM: diabetic rats. Lv: lentiviral vector. Data are mean ± SEM. *P < 0.01 vs. Con; &*P* > 0.05 vs. DM or DM + Lv-vehicle.

**Figure 2:**
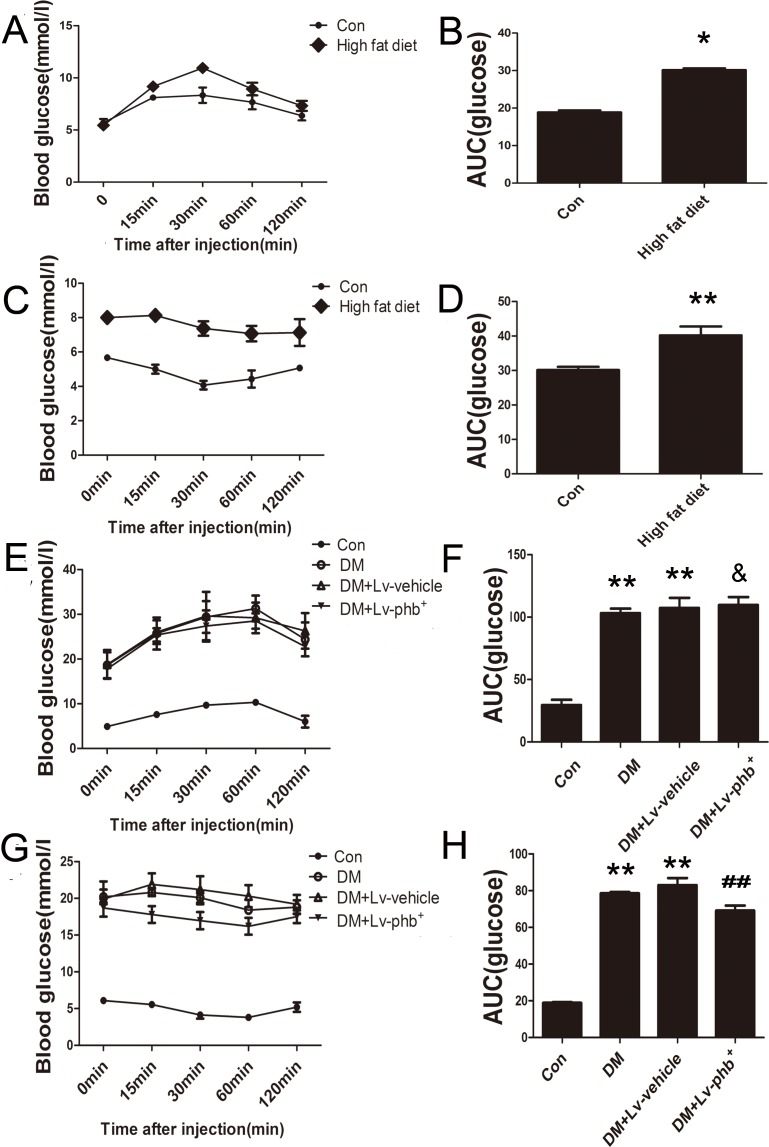
Intraperitoneal Glucose Tolerance Test (IPGTT) and Intraperitoneal Insulin Tolerance Test (IPITT) **A**. and **B**. Blood glucose and area under the receiver operating characteristic curve (AUC) for the IPGTT in rats at 4 weeks after induction of diabetes. **C**. and **D**. Blood glucose and AUC for the IPITT in rats at 4 weeks after induction of diabetes. (E and F) Blood glucose and AUC for the IPGTT in rats at 20 weeks after induction of diabetes. **G**. and **H**. Blood glucose and AUC for the IPITT in rats at 20 weeks after induction of diabetes. Con: normal rats, DM: diabetic rats, Lv: lentiviral vector. Data are mean ± SEM. *P < 0.01, **P < 0.05 vs. Con; & *P* > 0.05, ##*P* < 0.05 vs. DM or DM+Lv-vehicle.

After 4 weeks of a high-fat diet (HF; 34.5% fat, 17.5% protein, and 48% carbohydrate) diet, the mean area under the receiver operating characteristic curve (AUC) for intraperitoneal glucose tolerance test (IPGTT) and intraperitoneal insulin tolerance test (IPITT) was higher for HF diet rats than the controls (*P* < 0.01 and *P* < 0.05, respectively) (Figure 2A–2D). After 20 weeks of diabetes, diabetic rats showed significantly higher fasting blood glucose (FBG), total cholesterol (TC), and triglycerides (TG) levels (*P* < 0.01). PHB overexpression had no effect on the TC, TG and FBG (Figure 1). The AUC for IPITT was lower in rats overexpressing PHB than in vehicle-treated animals (*P* < 0.05) (Figure 2G, 2H).

### PHB overexpression attenuates diabetes-induced myocardial remodeling and cardiac dysfunction in rats

**Figure 3:**
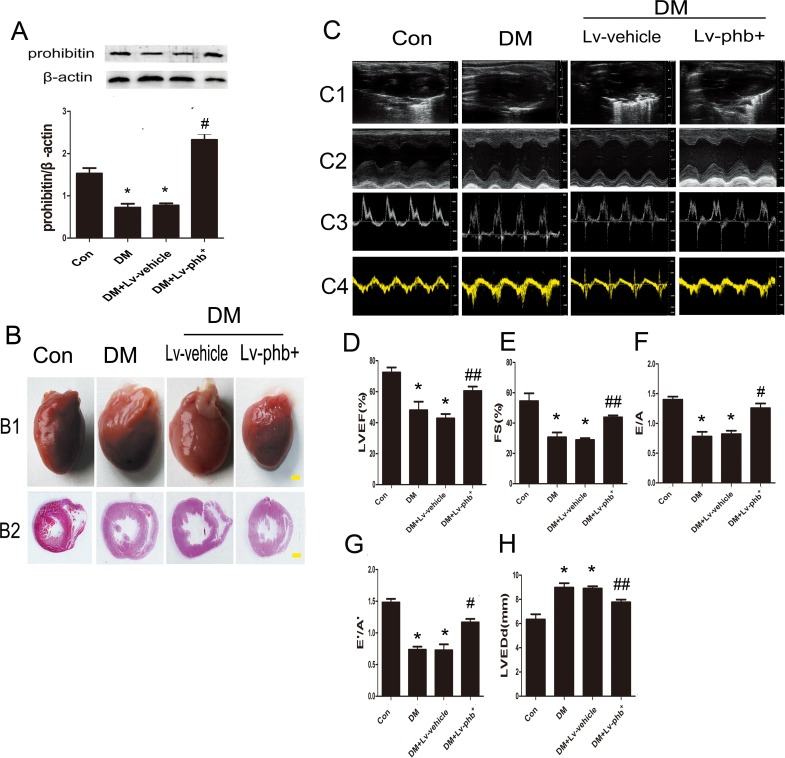
Prohibitin (PHB) expression improves cardiac dysfunction in diabetes rats **A**. Western blot analysis of PHB protein levels and quantitative analysis. **B1**. Heart sizes of the rats under different treatments (bar: 2mm). **B2**. Representative histological cross-sections of papillary muscle (bar: 2mm). **C1**. Representative 2D echocardiograms. **C2**. M-mode echocardiograms. **C3**. Pulsed-wave Doppler echocardiograms of mitral inflow. **C4**. Tissue Doppler echocardiograms. Quantitative analysis of LV ejection fraction (LVEF) **D**., fractional shortening (FS) **E**., early to late mitral flow (E/A) **F**., ratio of diastolic mitral annulus velocities (E'/A') **G**., and LV end-diastolic dimension (LVEDd) **H**. Con: normal rats, DM: diabetic rats, Lv: lentiviral vector. Data are mean ± SEM. *P < 0.01 vs. Con; #*P* < 0.01, ##*P* < 0.05 vs. DM or DM+Lv-vehicle.

We assessed whether PHB plays a role in myocardial fibrosis and diastolic dysfunction in type 2 diabetes. Diabetic rats showed significantly lower PHB protein levels in the heart than the controls (*P* < 0.01) (Figure 3A). The myocardial PHB protein level was higher in PHB-overexpressing than in vehicle-treated diabetic rats (*P* < 0.01) (Figure 3A). The DM group showed the phenotype of eccentric ventricular hypertrophy (Figure 3B).

We found that at 16 weeks after diabetes induction in rats. The cardiac function features of left ventricular (LV) ejection fraction (LVEF) and fractional shortening (FS) as well as the ratio of early to late mitral inflow velocity (E/A) and ratio of diastolic mitral annulus velocities (E'/A') ratios were lower in diabetic than in control rats (Both P<0.01) (Figure 3D–3G). In contrast, LV end-diastolic dimension (LVEDd) was higher in diabetic rats than in controls (*P* < 0.01) (Figure 3H). When compared with vehicle, increased LVEF, FS, E/A, E'/A' and decreased LVEDd were observed in the PHB-overexpressing group (*P* < 0.05 or *P* < 0.01) (Figure 3C–3H). The blood pressure of diabetic rats was slightly increased, but no statistical significance was found (Table 1).

**Table 1:** Blood pressure in rats with various treatments

	SPB (mmHg)	MBP (mmHg)	DPB (mmHg)
Con	117.7 ± 3.8	99.9 ± 4.4	91.1 ± 5.6
DM	120.7 ± 4.9	102.7 ± 3.8	93.8 ± 5.5
DM+Lv-vehicle	121.0 ± 5.4	103.1 ± 4.6	94.2 ± 5.4
DM+Lv-phb+	118.0 ± 2.5	100.8 ± 4.2	92.2 ± 6.2

Blood pressures were measured at 16 weeks after diabetes. SBP, systolic blood pressure; MBP, mean blood pressure; DBP, diastolic blood pressure. Con: normal rats, DM: diabetic rats, Lv: lentiviral vector. Data are the mean ± SEM.

### PHB overexpression alleviates diabetes-induced myocardial fibrosis and apoptosis *in vivo*

**Figure 4:**
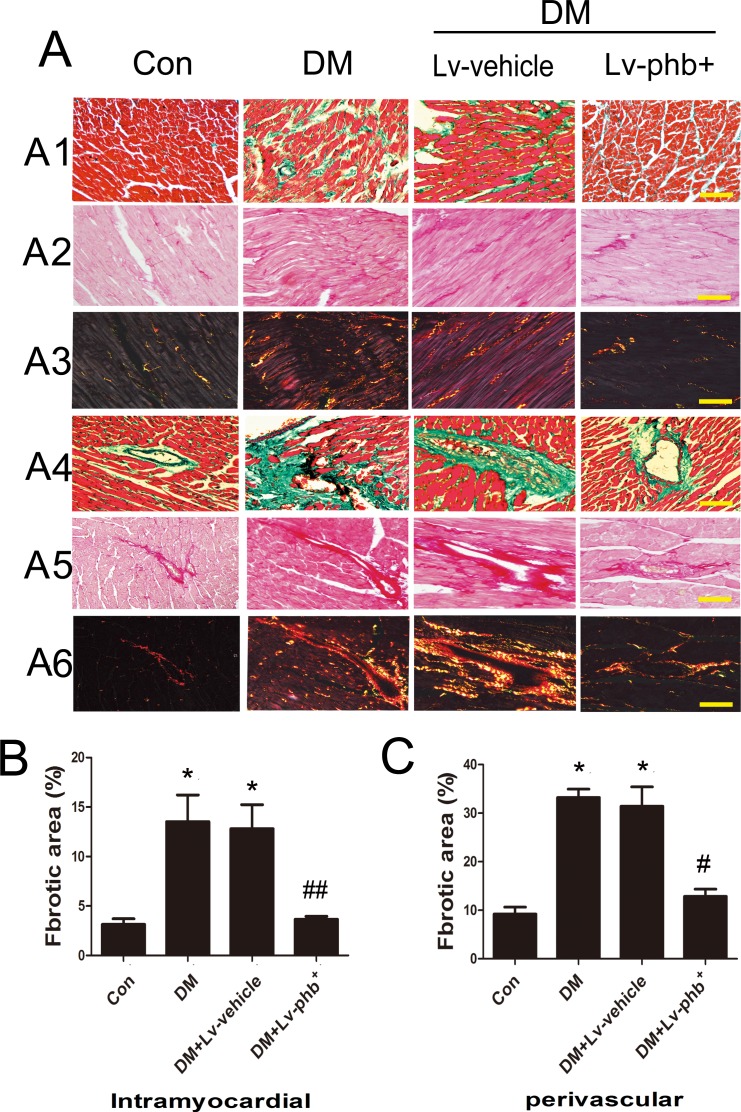
Effect of PHB on collagen deposition **A1**. Masson'strichrome staining of the intramyocardial area of rat heart (bar: 100μm). **A2**.–**A3**. Picrosirius red staining of the intramyocardial area (bar: 100μm). **A4**. Masson'strichrome staining of perivascular area (bar: 100μm). **A5**.-**A6**. Picrosirius red staining of the perivascular area (bar: 100m). **B**. Fibrotic area of the intramyocardial area. **C**. Fibrotic area of the perivascular area. Con: normal rats, DM: diabetic rats, Lv: lentiviral vector. Data are mean ± SEM. *P < 0.01 vs. Con; #*P* < 0.01, ##*P* < 0.05 vs. DM or DM+Lv-vehicle.

**Figure 5:**
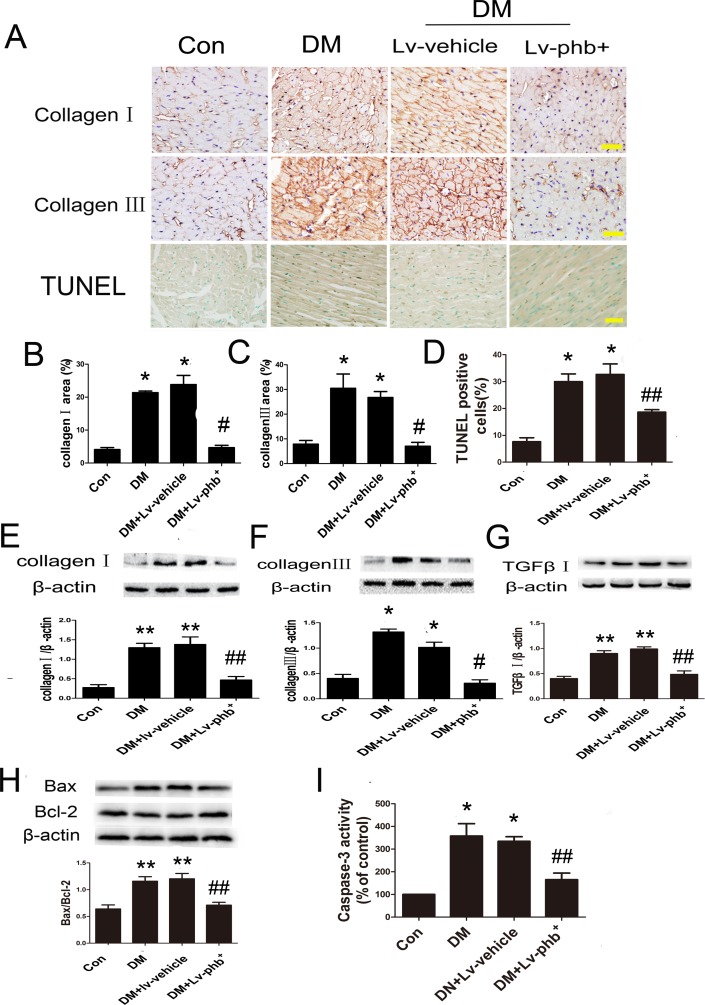
Effect of PHB on collagen deposition and apoptosis *in vivo* **A**. Immunostaining of collagen I (upper panels), collagen III (middle panels), Terminal Deoxynucleotidyl Transferase dUTP Nick End Labeling (TUNEL) staining of myocardium (lower panels) (bar: 50μm) and quantification of collagen I area **B**., collagen III area **C**., and apoptosis rate **D**. **E**.–**G**. Western blot analysis and quantification of protein expression of collagen I and III and TGF-β1 in rats. **H**. Western blot analysis and quantification of Bcl2-associated X protein (Bax) and B-cell leukemia/lymphoma-2 (Bcl-2). **I**. Quantification of caspase-3 activity as % of control. Con: normal rats or normal glucose (NG: 5.5mM), DM: diabetic rats, Lv: lentiviral vector. Data are mean ± SEM. *P < 0.01, **P < 0.05 vs. Con; #*P* < 0.01, ##*P* < 0.05, vs. DM or DM+Lv-vehicle.

Masson's trichome and Picrosirius red staining of heart sections demonstrated increased ECM in the interstitial regions of the diabetic myocardium compared to controls (Figure 4A). Diabetic rats showed increased collagen deposition in intramyocardial areas and perivascular areas compared to controls (Both *P* < 0.01) (Figure 4B, 4C). PHB overexpression reduced the collagen deposition as compared to vehicle treatment (*P* < 0.05, intramyocardial; *P* < 0.01, perivascular) (Figure 4B, 4C). Diabetes increased the expression of fibrotic markers collagen I and III compared to controls, while PHB overexpression significantly reduced the levels compared to vehicle treatment as indicated by immunohistochemistry (*P* < 0.01 for both collagen I and III) (Figure 5A–5C) and western blot analysis (*P* < 0.05 or P < 0.01) (Figure 5E–5G).

Diabetes significantly increased myocardial caspase-3 activity (*P* <0.01) and the ratio of Bax/Bcl-2 (*P* < 0.05 or *P* < 0.01) (Figure 5H, 5I). In addition, the proportion of Terminal Deoxynucleotidyl Transferase dUTP Nick End Labeling (TUNEL)-positive apoptotic cells was significantly increased in diabetic hearts (*P* < 0.01) (Figure 5D). PHB effectively ameliorated hyperglycemia-activated caspase-3 and decreased Bax/Bcl-2 ratio (Both *P* < 0.05) (Figure 5H, 5I). Additionally, the proportion of TUNEL-positive cells in the diabetic mouse also decreased by PHB overexpression (*P* < 0.05) (Figure 5D).

### PHB is located in the cytoplasm and nucleus in CFs and PHB overexpression inhibits CF proliferation

**Figure 6:**
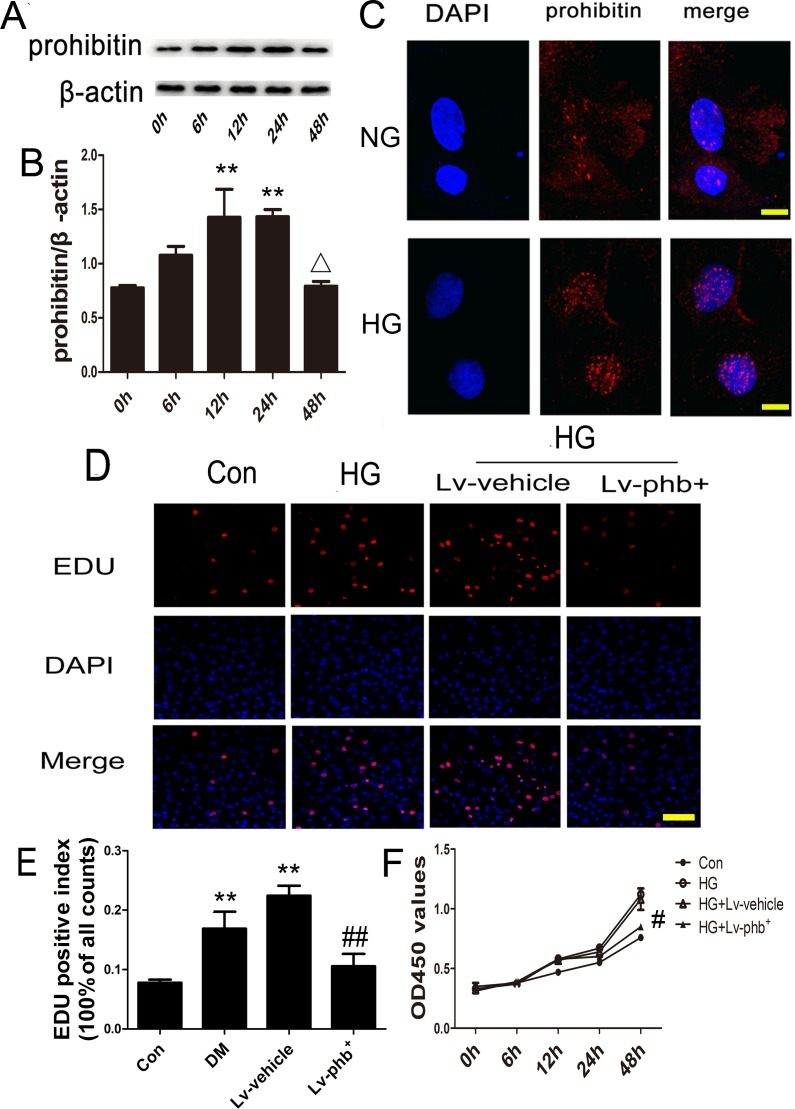
HG effect on PHB production in CFs and PHB overexpression inhibiting CF proliferation **A**. Cardiofibroblasts (CFs) were stimulated with HG for various periods; western blot analysis of protein expression of PHB, and **B**. quantification. **C**. Immunocytochemistry for PHB localization after NG and HG treatment (bar: 10μm). PHB is stained red; nuclei were counterstained with DAPI (blue). **D**. Laser confocal microscopy of 5-ethynyl-2-deoxyuridine (EdU) staining (bar: 100μm). **E**. EdU-positive index expressed as % of cell counts. **F**. Cell Counting Kit-8 analysis the cell viability of CFs treated with glucose for various times. Con: normal glucose (NG: 5.5mM); HG: 30mM glucose, Lv: lentiviral vector. Data are mean ± SEM. **P < 0.05 vs. Con; Δ*P* < 0.05 vs. 24 h. #*p*<0.01, ##*P* < 0.05 vs. HG or HG+Lv-vehicle.

Exposure of the CFs to high glucose (HG; 30 mM) for various periods showed that the PHB protein level increased during the first 24 h (*P* < 0.05), while it decreased at 48 h (*P* < 0.05) (Figure 6A, 6B). PHB was located in the cytoplasm and nucleus after normal glucose (NG; 5.5 mM) and HG treatment. The nuclear expression of PHB increased after HG treatment (Figure 6C).

Because CF proliferation plays an important role in myocardial fibrosis, we examined the effect of PHB on CF proliferation. HG treatment increased the growth rate of the CFs, while PHB overexpression inhibited the HG-induced increase in cell proliferation (*P* < 0.05) (Figure 6D–6F).

### PHB overexpression reduces collagen I, III, MMP-2 and MMP-9 expression induced by HG in CFs

**Figure 7:**
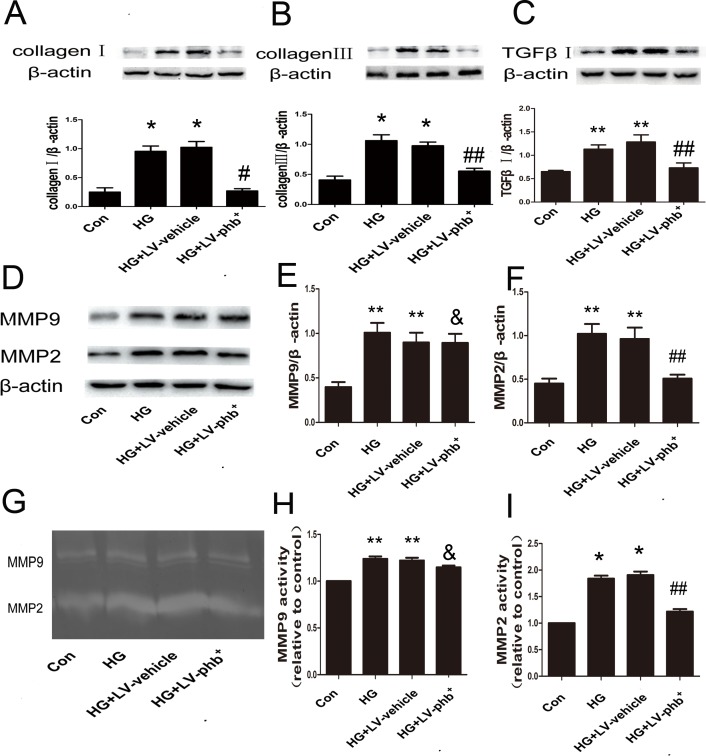
Effect of PHB on protein expression of collagen and MMPs in CFs **A**.–**C**. Western blot analysis of protein expression of collagen I and III and TGF-β1 in CFs. **D**. Western blot analysis of matrix metalloproteinase 9 (MMP-9) and MMP-2 protein levels and **E**., **F**. quantification. **G**. Gelatin zymography of activity of MMP-2 and MMP-9 and **H**., **I**. quantification. Con: normal glucose (NG: 5.5mM), HG: 30mM glucose, Lv: lentiviral vector. Data are mean ± SEM. *P < 0.01, **P < 0.05 vs. Con; &*P* > 0.05, #*p*<0.01, ##*P* < 0.05, vs. HG or HG+Lv-vehicle.

In CFs induced with HG for 48 h, the levels of TGF-β1 (*P* < 0.05), and collagen I and III (Both *P* < 0.01) were increased; PHB overexpression attenuated the increase of all three proteins (*P* < 0.05 or *P* < 0.01) (Figure 7A–7C).

HG increased matrix metalloproteinase 2 (MMP-2) and MMP-9 expression (Both *P* < 0.05) (Figure 7D–7F) and activity (*P* < 0.05 or *P* < 0.01) (Figure 7G–7I) in the CFs as revealed by zymography. PHB overexpression markedly abrogated the HG-induced increase in activity of MMP-2 (P < 0.05), but not that of MMP-9 (Figure 7G–7I), which was confirmed by the western blot results (Figure 7D–7F).

### PHB overexpression decreases HG-induced ROS production and apoptosis of H9c2 cardiomyoblasts

**Figure 8:**
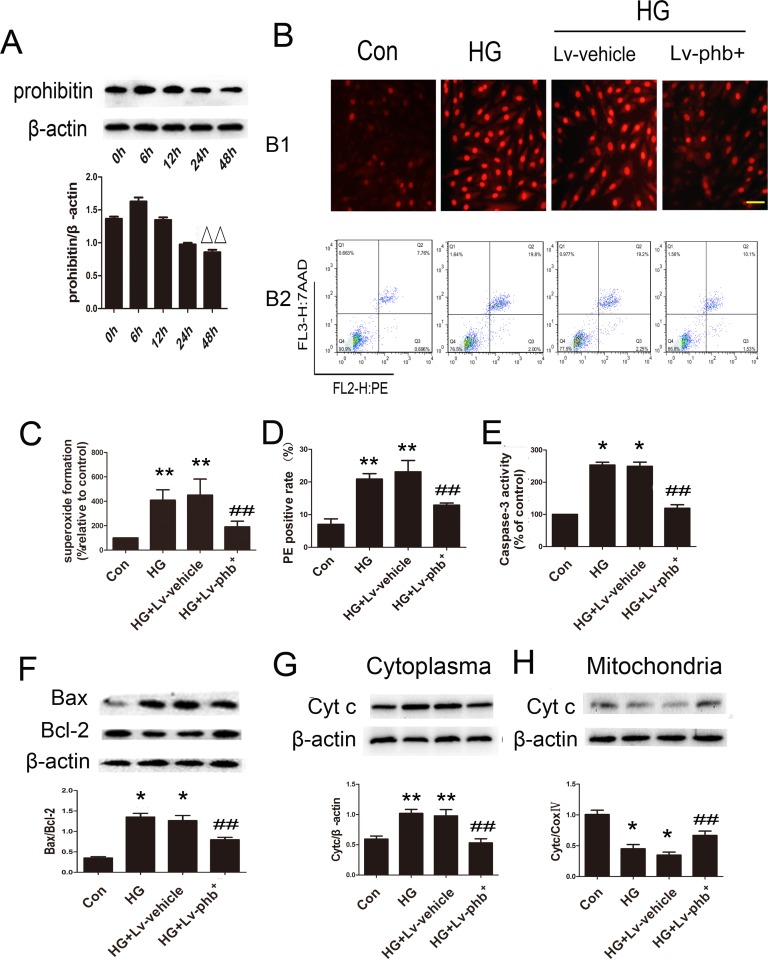
Role of PHB in ROS accumulation and apoptosis **A**. H9c2 cardiomyoblasts were stimulated with HG for various periods; western blot analysis of protein expression of PHB and quantification. **B1**. Dihydroethidium (DHE) staining of H9c2 cardiomyoblasts and **C**., quantification (bar: 50μm). **B2**. Flow cytometry with phycoerythrin(PE)/7-amino-actinomycin D (7-AAD) staining to determine cell apoptosis. **D**. Quantitative analysis of PE positive rate. **E**. Quantification of caspase-3 activity as % of control. **F**. Western blot analysis and quantification of Bax and Bcl-2. **G**. and **H**. Western blot analysis and quantification of cytochrome c. Con: normal rats or normal glucose (NG: 5.5mM), DM: diabetic rats, HG: 30mM glucose, Lv: lentiviral vector. Data are mean ± SEM. ΔΔ*P* < 0.05 vs. 6 h. *P < 0.01, **P < 0.05 vs. Con. ##*P* < 0.05 vs. HG or HG+Lv-vehicle.

Exposure of the H9c2 cardiomyoblasts to HG for various periods showed that the PHB protein level decreased significantly at 48h compared to 6h (*P* < 0.05) (Figure 8A). Dihydroethidium (DHE) staining and flow cytometry were used to examine the role of PHB in H9c2 cardiomyoblasts. HG significantly increased the ROS level while PHB overexpression attenuated ROS production (*P* < 0.05) (Figure 8B1, 8C). PHB overexpression reduced the proportion of phycoerythrin (PE)-positive apoptotic cells induced by HG (*P* < 0.05) (Figure 8B2, 8D).

HG increased the level of caspase-3 activity and Bcl2-associated X protein/B-cell leukemia/lymphoma-2 (Bax/Bcl-2) ratio (Figure 8E, 8F) as well as the mitochondrial cytochrome c protein level compared to that in the cytoplasm (Figure 8G, 8H). PHB overexpression attenuated the HG-induced apoptotic effect (Both *P* < 0.05).

### PHB overexpression mitigates HG-induced ERK and Akt activation in myocardium

**Figure 9:**
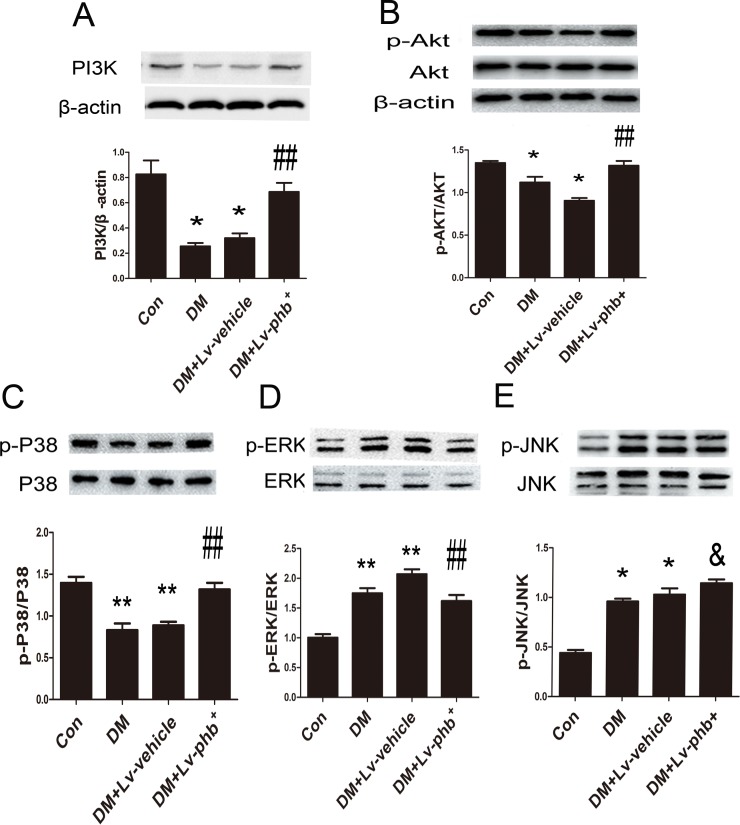
Signal-transduction mechanisms in PHB functioning in myocardium **A**.-**E**. Western blot analysis of PI3K, p-Akt, phospho-extracellular signal-regulated kinase (p-ERK), p-p38 and p-Jun NH2-terminal kinase (p-JNK). Con: normal rats, DM: diabetic rats. Lv: lentiviral vector. Data are mean ± SEM. *p < 0.01, **p < 0.05 vs. Con; & *P* > 0.05, ##*P* < 0.05 vs. DM or DM+Lv-vehicle.

To clarify the mechanism underlying PHB function further, we examined the potential signal transduction pathways involved in diabetes-induced myocardial fibrosis. The PI3K, p-Akt and p-P38 levels were decreased in diabetic rats, and PHB overexpression increased the levels compared to vehicle treatment (Both *P* < 0.05) (Figure 9A–9C). PHB overexpression reduced diabetes-induced phosphorylation of extracellular signal-regulated kinase 1/2 (ERK1/2) in the myocardium *in vivo* (*P* < 0.05) (Figure 9D).

## DISCUSSION

Myocardial damage induced by type 2 diabetes is associated with insulin resistance, oxidative damage, cardiocyte apoptosis, and interstitial fibrosis [19–21]. Hyperglycemia, altered insulin action, and increased levels of nonesterified fatty acids may trigger the cardiac phenotype in type 2 diabetes [22]. PHB is an intracellular protein with antiproliferative activity [23]. Furthermore, it has anti-inflammatory activity and protects against oxidative stress-induced cell injury [24]. However, its function in DCM is still unknown. The present study showed that in type 2 diabetic rats with insulin resistance, PHB overexpression ameliorated LV dysfunction, cardiac remodeling, improving insulin resistance, and alleviated collagen deposition and apoptosis both *in vitro* and *in vivo*. To our knowledge, this is the first report demonstrating the protective role of PHB in DCM.

Intensive cardiac fibrosis, which can induce LV stiffness, is a common finding in progressed DCM [4]. Excess production of ECM, mainly collagen types I and III, can alter the structure and function of the heart and is one of the most important pathological features of DCM [25]. TGF-β1 is also overexpressed in DCM and is involved in fibrotic processes [26]. Our study revealed that overexpression of PHB reduced aberrant interstitial and perivascular deposition of collagen in interstitial areas on Masson and Sirius red staining, as well as expression of collagen I and III and TGF-β1. Consistent with this, echocardiography revealed cardiac dysfunction. Fibroblasts could provide structural support by balance ECM deposition and turnover [27, 28]. A previous study has proven that MMP2 stimulates collagen I expression in rat CFs and activation of MMPs is associated with cardiac fibrosis [29]. Indeed, we found that HG stimulated MMP-2 and MMP-9 expression/activation in the CFs, concomitant with increased collagen expression *in vitro*, while PHB overexpression reduced the activity and expression of MMP-2. What's more, PHB overexpression significantly inhibited the proliferation of CFs induced by HG, which may contribute to ameliorating altered cardiac function in diabetes.

Continuous loss of cardiomyocytes is one of the hallmarks of myocyte hypertrophy and fibrosis [30, 31]. Suppression of cardiomyocyte apoptosis prevents the development of myocardiopathy [32, 33]. As we expect, PHB overexpression significantly decreased the apoptosis in both H9c2 cardiomyoblasts and diabetic rats. The ratio of anti- and pro-apoptotic proteins determines cell survival or apoptosis treated with an apoptotic stimulus [34]. PHB reduced HG-induced caspase-3 activity and the Bax/Bcl-2 ratio in both HG-stimulated cardiomyocytes and diabetic hearts, this is in accordance with previous study that the expression of Bcl-2 reduced and Bax is enhanced in cardiomyocytes under HG conditions [35].

Under HG or hyperglycemia, cardiomyocytes release cytokines and produce ROS, which trigger cell-death signaling cascades [36, 37]. It has been reported that PHB overexpression protect the mitochondria from apoptosis by suppressing oxidative stress-induced injury and reverse AngII induced cardiomyocyte hypertrophy. In our study, PHB overexpression significantly reduced HG-induced intracellular ROS and inhibited the release of cytochrome c from mitochondria to the cytosol, resulting in suppression of HG-induced cardiomyocyte apoptosis.

We further studied the molecular mechanism of PHB *in vivo*. Wang et al. found that miR-361 regulated prohibitin expression and was involved in the regulation of mitochondrial network in cardiomyocytes [40]. In our study, after miR-361 antagomir transfection under the treatment of high glucose for 48 h, the level of PHB protein expression of H9c2 cardiomyoblasts was significantly increased. The result was shown in Supplementary Figure S1A, which will further strengthen our results.

The insulin-Akt signaling pathway is involved in the progression of DCM [41–43]. PHB can interact with phosphatidylinositol 3,4,5-triphosphate (PIP3)/Akt and modulate insulin signaling [44]. What's more, Kuo et al. reported that insulin-like growth factor 1(IGF-I)/IGF-IR exert an anti-apoptotic effect via PI3k and Akt-dependent pathway [45]. To clarify whether PHB resulted apoptosis inhibition is mediated by activating this pathway, the levels of PI3k and Akt were analyzed. In our study, the Akt pathways appeared to be selectively inhibited, while PHB overexpression increased the phosphorylation of Akt, which may contribute to the improved insulin resistance and decreased apoptosis rate of cardiomyocytes.

The major MAPK signaling cascades ERK1/2, p38, and JNK are strongly activated by HG [46]. They participated in the progression of cellular hypertrophy, apoptosis, cardiac fibrosis, and cardiac cytokine-mediated inflammation, which were involved in the development of DCM [47]. Previous studies showed that H2S could prevent HG-induced H9C2 cells apoptosis via inhibiting the activation of the ERK1/2 pathway [48]. HMGB1 mediates hyperglycemia-induced cardiomyocyte apoptosis via ERK/Ets-1 signaling pathway [49]. Thus, the decreased p-ERK1/2 protein level by PHB overexpression may protect the DCM. In contrast, PHB elevated the level of p-P38 that decreased in diabetes, which is in line with previous findings that inhibition of p-P38 results in superinduction of procollagen type I expression in both rigid and mechanically loaded cardiac fibroblasts [50].

In conclusion, we showed that PHB protects against DCM in a rat model. The PHB effect was associated with the Akt and MAPK signal pathway and alleviated accumulation of ECM in the diabetic cardiac interstitium and apoptosis of cardiomyocytes. However, the exact underlying mechanisms are not fully understood. The H9C2 cell was derived from the embryonic rat ventricle and is being a surrogate for cardiomyocytes, it has the characteristics of both skeletal and cardiac muscle cells [51]. Although it has been proven by some studies that it's an excellent *in vitro* model system for prospective molecular studies in heart development and disease, the extent to which H9C2 cells can accurately mimic the responses of primary cardiac myocytes has not yet been fully established [52], further investigations using cell culture of cardiomyocytes and inhibition of signaling pathways should unravel these mechanisms.

## MATERIALS AND METHODS

### Animal model

Sixty male Sprague-Dawley rats (120–140g) were purchased from Beijing Weitong Lihua Experimental Animal Technology (Beijing, China). The animals were housed at 22°C with a 12-h light/dark cycle. After 1 week of acclimatization, IPGTT was performed. The rats were randomly divided into 4 groups: healthy control (Con; n = 15), diabetes alone (DM; n = 15), diabetes + lentivector-vehicle (DM + Lv-vehicle; n = 15), and diabetes + lentivector-PHB (DM + Lv-PHB^+^; n = 15). The 3 groups of diabetic rats were fed a HF diet for 4 weeks. Then, the IPGTT was repeated and IPITT was performed, and blood was sampled through the angular vein. Diabetes was induced by a single intraperitoneal injection of streptozotocin (STZ; Sigma, St. Louis, MO; 27.5 mg/kg in 0.1 mol/L citrate buffer, pH 4.5) in rats with insulin resistance [53]. Rats with FBG > 11.1 mmol/L in 2 consecutive analyses at 1 week after STZ administration were considered the type-2 diabetic model rats. At 12 weeks after diabetes induction, 2 groups of diabetic rats were injected with 5×10^7^ UT/50 uL lentiviral vector carrying PHB cDNA-GFP (GenePharma, Shanghai, China) or the same volume of vehicle lentiviral vector (GenePharma, Shanghai, China) via the jugular vein. At 16 weeks after diabetes induction, rats were sacrificed. Serum cholesterol, triglyceride levels, and FBG were analyzed by the Bayer 1650 blood chemistry analyzer (Bayer, Tarrytown, NY). The IPGTT, IPITT, and blood analyses was performed as described [53]. All experiments conformed to the Guide for the Care and Use of Laboratory Animals published by the US National Institutes of Health and Shandong University. The study protocol was approved by the Institutional Ethics Committee of Shandong University.

### Cardiac function measurement

According to previous studies, beginning from 2 to 3 months after the induction of diabetes, diabetic rats showed onset of cardiac dysfunction [53, 54]. At 16 weeks after STZ injection, blood pressure was measured by BP-98A computerized tail-cuff system (Softron, Tokyo) using the tail cuff method in conscious rats. Transthoracic echocardiography was performed as described [54].

### Histology and immunohistochemistry

Rat hearts fixed in 4% paraformaldehyde were bisected transversely at the midventricular level, embedded in paraffin, and cut into 4mm sections for staining with hematoxylin and eosin (H&E). Additionally, heart sections were stained with Masson's trichrome and Picrosirius red to examine ECM deposition. For immunohistochemistry, In brief, after blocking, sections were incubated with primary antibody overnight at 4°C, then washed with phosphate buffered saline and secondary antibody at 37°C for 30 min.

### TUNEL staining

Apoptosis was detected by using a commercial DNA fragmentation detection kit (Millipore, Billerica, MA, USA) according to the manufacturer's instructions.

### Cell culture and treatment

CFs were isolated from neonatal rat ventricular tissues [18]. CFs and H9c2 cardiomyoblasts at 60% confluence were exposed to HG or NG. The cells were cultured in 6-well culture plates and infected with recombinant lentiviruses at a multiplicity of infection (MOI) 50. After 24h, the medium was replaced with fresh complete medium. The cells were further cultured for 48h before observation under a fluorescence microscope to confirm that more than 90% cells were positive for GFP (Supplementary Figure S2A).

### Immunofluorescence microscopy

In brief, after blocking, cells were incubated with primary antibody at 4°C overnight, then washed with phosphate buffered saline and fluorescent dye-conjugated secondary antibody for 30 min at 37°C.

### Western blot analysis

Whole-cell proteins were isolated from cell lysates and freshly dissected rat hearts. Mitochondrial proteins of H9c2 cardiomyoblasts were isolated by using a commercial cell mitochondria isolation kit (Beyotime, China). Western blot analysis was performed as described [55]. The membans were incubated overnight with rabbit primary antibodies against PHB, collagen I and III, TGF-β1, MMP-2 and MMP-9, Bcl-2, Bax, cytochrome c, Cox IV(Abcam, Cambridge, MA), PI3K, p-Akt (ser473)/Akt, p-p38 (Thr180/Tyr182)/p38 MAPK, p-ERK (Thr 202/Tyr 204)/ERK, p-Jun NH2-terminal kinase (p-JNK) (Thr183/Tyr185)/JNK (Cell Signaling Technology, Beverly, MA).

### Gelatin zymography

The activity of MMP-2 and MMP-9 was measured by gelatin zymography as described [56].

### Assessment of cell proliferation

CFs were cultured in 96-well culture plates and infected with recombinant lentiviruses. Cell proliferation was determined at 0, 6, 12, 24 or 48h after transfection by using the Cell Counting Kit-8 (Dojindo Molecular Technologies, Kumamoto, Japan). The opticaldensity (OD) at 450nm was determined using a spectrophotometer. The Cell-Light 5-ethynyl-2′-deoxyuridine (EdU) kit (Rib Bio, Suzhou, China) was used according to the manufacturer's instructions to label proliferating cells.

### Intracellular ROS detection

H9c2 cardiomyoblasts were incubated with a DHE solution (10μM) (Beyotime, Beijing) at 37°C for 2h. Then, the solution was replaced with PBS and the cells were observed under the fluorescence microscope.

### Analysis of apoptosis with flow cytometry

H9c2 cardiomyoblasts were collected and suspended in 100μL binding buffer. Five microliters of Annexin V-PE and 5μL of 7-amino-actinomycin D (7-AAD) were added to each sample and the mixture was incubated in the dark at room temperature for 15min. Apoptotic cells stained positive for Annexin V-PE.

### Caspase-3 activity assay

The colorimetric assay (Beyotime, China) was used to measure caspase-3 activity. It was performed according to the manufacturer.

### Statistical analysis

Data are reported as the mean ± SEM. Results were compared by 2-tailed Student's t test for 2 groups and one-way ANOVA followed by the Tukey's t test (2-tailed) for multiple groups. SPSS v16.0 (SPSS Inc., Chicago, IL) was used for analysis. Differences were considered statistically significant at P < 0.05.

## SUPPLEMENTARY MATERIAL FIGURES


