# Supplementary material for: Prohibitin overexpression improves myocardial function in diabetic cardiomyopathy
Source: Oncotarget. 2015 Nov 25;7(1):66–80. doi: 10.18632/oncotarget.6384 (PMC4807983; doi:10.18632/oncotarget.6384)
Supplement: Supplementary file 1 [file oncotarget-07-0066-s001.pdf]

Prohibitin overexpression improves myocardial function in diabetic cardiomyopathy

Supplementary Material

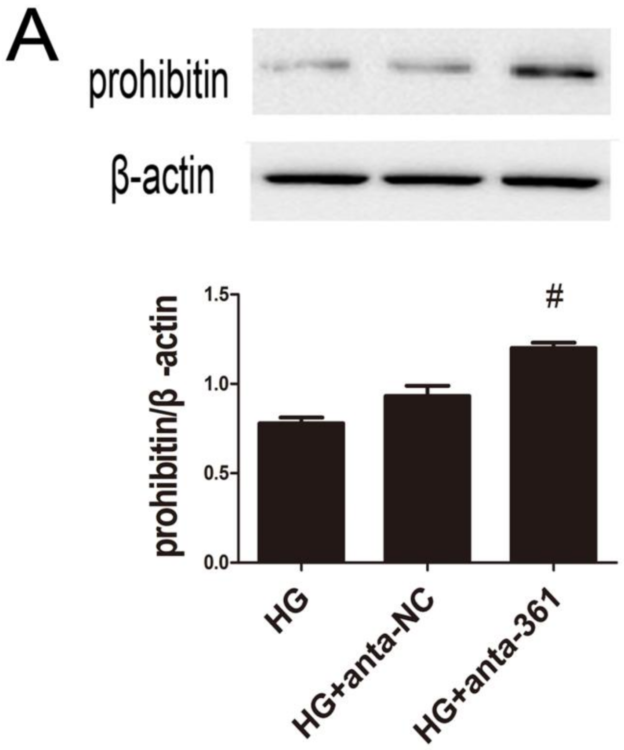

Supplementary Figure S1. miR-361 Regulate the Expression of PHB.

(A) Western blot analysis of the PHB protein level after transfection of miR-361 antagomir under the treatment of high glucose for 48 in H9c2 cardiomyoblasts. The miR-361 antagomir sequence was 5′ -GUACCCCUGGAGAUUCUGAUAA-3. The antagomir-NC sequence was 5′ -CAGUACUUUUGUGUAGUACAA-3. Con: normal rats, DM: diabetic rats, HG: 30mM glucose, Lv: lentiviral vector. Data are mean ± SEM. #p<0.01 vs. HG or HG+anta-NC.

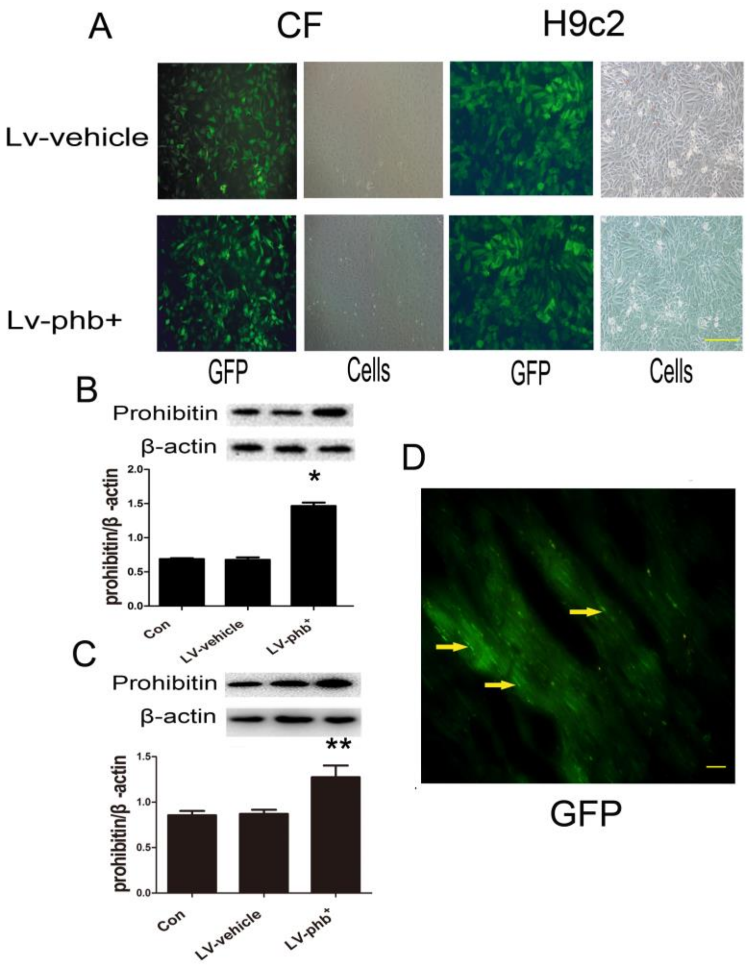

Supplementary Figure S2. Overexpression of PHB in Cardiofibroblasts by Lentivirus Transfection.

(A and B) Representative photograph of GFP-labeled scramble transfection efficiency in cardiofibroblasts and H9c2 cardiomyoblast by fluorescence microscopy; the transfection efficiency was evaluated more than 90% (bar: 200μm). **(B)** Western blot analysis of the PHB protein level relative to that of β-actin in cardiofibroblasts and quantitative analysis. **(C)** Western blot analysis of the PHB protein level relative to that of β-actin in H9c2 cardiomyoblas and quantitative analysis. (**D)** Representative fluorescence microscopy of GFP-labeled scramble transfection efficiency in rat myocardial tissue (bar: 20μm). Con: normal rats, Lv: lentiviral vector. Data are mean ± SEM. *P < 0.01, ** P < 0.05 vs. Con or Lv-vehicle.

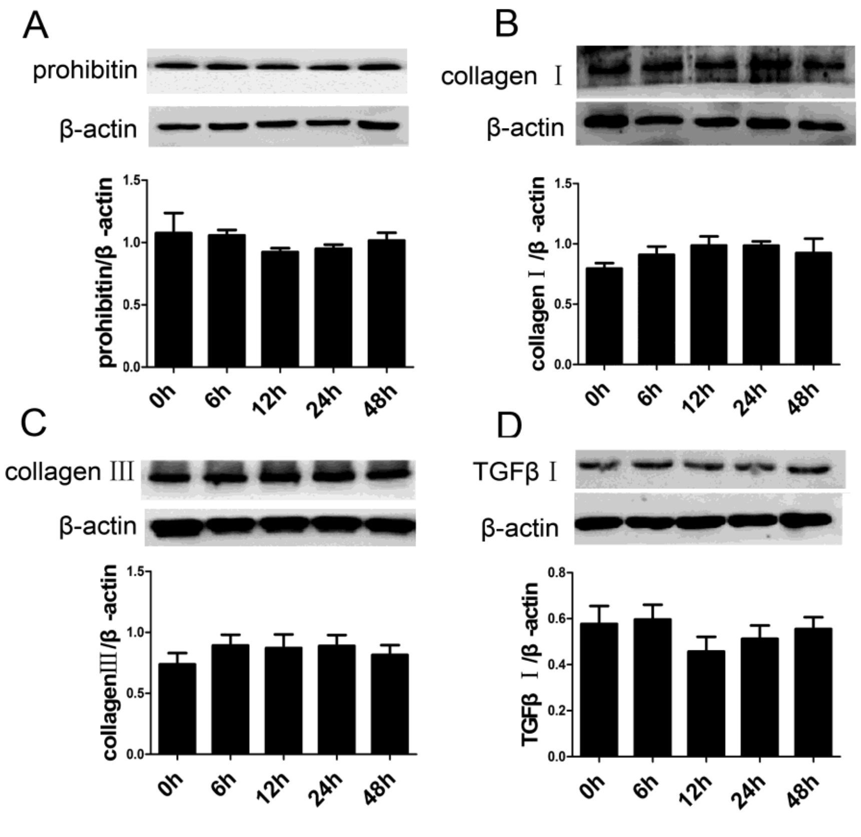

Supplementary Figure S3. Osmotic Pressure's Effect on CFs

(A–D) Western blot analysis of protein expression of PHB, collagen I and III and TGF-β1 in CFs with OC (5.5 mmol/l glucose plus 24.5 mmol/l mannose) treatment for various periods.

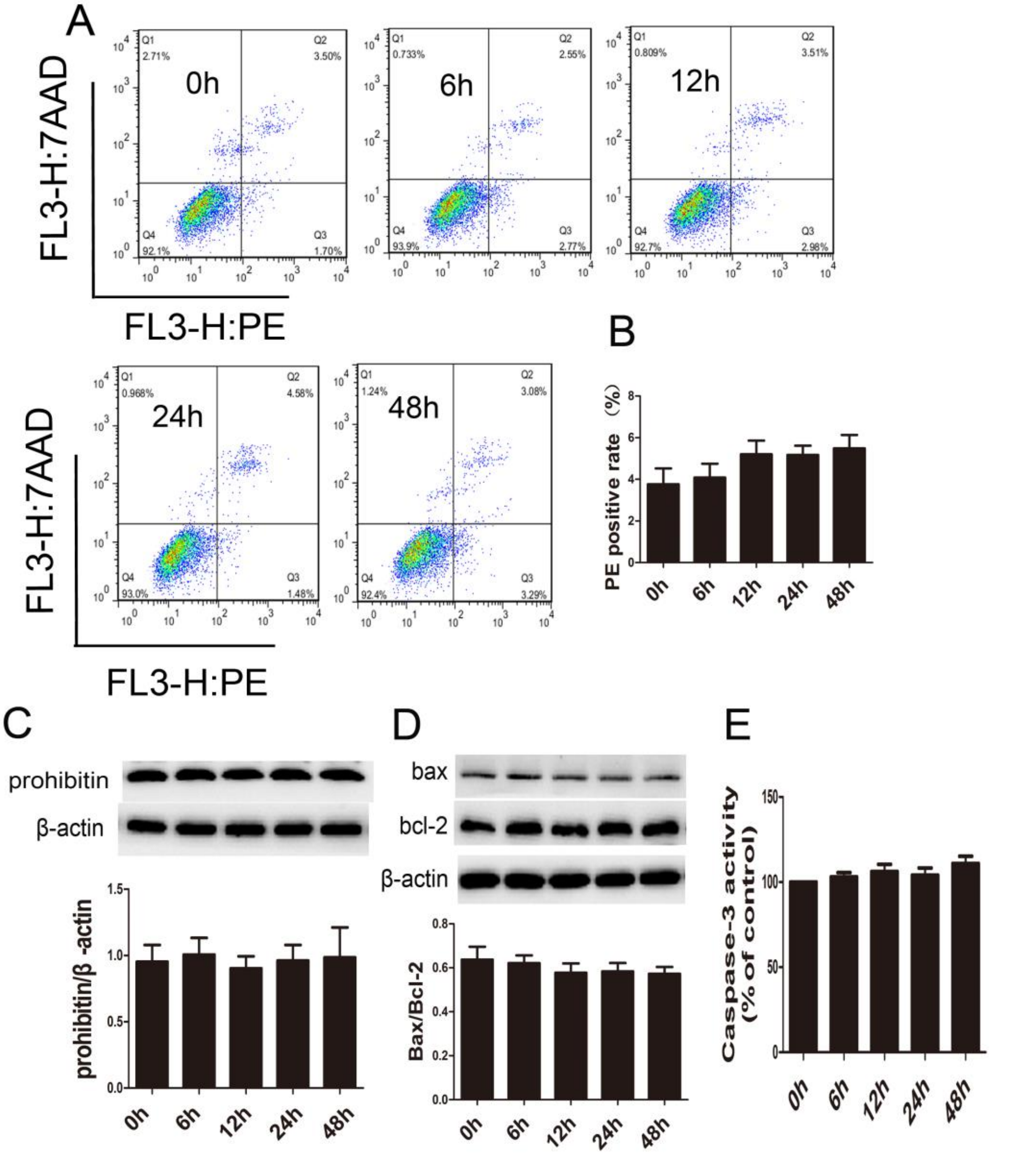

Supplementary Figure S4. Osmotic Pressure's Effect on Cardiomyoblasts.

After treated with OC (5.5 mmol/l glucose plus 24.5 mmol/l mannose) treatmen for various periods **(A)** Flow cytometry with phycoerythrin(PE)/7-amino-actinomycin D (7-AAD) staining to determine cell apoptosis. **(B)** Quantitative analysis of PE positive rate. **(C–D)** Western blot analysis and quantification of PHB, Bax and Bcl-2. **(E)** Quantification of caspase-3 activity as % of control.
